# The Effect of the Experimental Training Program ‘Grappler Quest’ on the Motor Fitness of Brazilian Jiu-Jitsu Athletes

**DOI:** 10.3390/jcm15135176

**Published:** 2026-07-02

**Authors:** Wojciech Wąsacz, Łukasz Rydzik, Tomasz Pałka, Paweł Ostrowski, Tadeusz Ambroży

**Affiliations:** 1 Faculty of Physical Education and Sport, Institute of Sports Sciences, University of Physical Culture in Krakow, 31-571 Krakow, Poland; lukasz.rydzik@awf.krakow.pl (Ł.R.); tadeusz.ambrozy@awf.krakow.pl (T.A.); 2 Department of Physiology and Biochemistry, Faculty of Physical Education and Sport, University of Physical Culture in Krakow, 31-571 Krakow, Poland; tomasz.palka@awf.krakow.pl; 3 Institute of Physical Culture Studies, College of Medical Sciences, University of Rzeszow, 35-310 Rzeszow, Poland; ostrowskipawel@onet.pl

**Keywords:** combat sports, flagship ground-based grappling, experimental intervention, circuit training, motor fitness, randomised trial

## Abstract

**Featured Application:**

The present findings suggest that structured circuit-based exercises may represent a valuable complement to standard Brazilian Jiu-Jitsu training by promoting improvements in selected components of motor fitness, particularly strength endurance. The practical relevance of Grappler Quest should be interpreted primarily in athletic conditioning and motor-preparation contexts. Any implications for training safety, injury-risk reduction, or return-to-training procedures remain indirect and require confirmation in prospective studies, including injury surveillance, rehabilitation outcomes, and return-to-play measures.

**Abstract:**

**Background/Objectives**: Despite the growing popularity of specialised training interventions aimed at developing motor abilities relevant to combat sports, scientific evidence regarding their effectiveness in Brazilian Jiu-Jitsu (BJJ) remains limited. This study aimed to estimate the effects of the experimental Grappler Quest (GQ) training program on the motor fitness profile of BJJ athletes and to explore whether training experience was associated with the magnitude of training-related changes. **Methods**: In this randomised trial, 44 competitive male BJJ athletes were allocated to an experimental group (EXP; *n* = 22) or a control group (CON; *n* = 22). Both groups followed an 8-week training protocol: the EXP group performed the structured GQ program, consisting of small circuit-based workouts, including resistance, plyometric, gymnastic, and BJJ-related exercises, whereas the CON group followed a standard BJJ training cycle. The motor profile was assessed before and after the intervention (pretest vs. posttest) using selected motor tests. The prespecified primary outcomes were strength-endurance performance in the bench press and squat performed with 50% body mass. Other motor-performance outcomes were treated as secondary or exploratory. Associations between training experience and intervention-related changes were analysed exploratorily. **Results**: ANCOVA of adjusted post-intervention means indicated between-group differences favouring the EXP group. Large effects were observed for the primary strength-endurance outcomes: bench press at 50% body mass (η^2^ = 0.52) and squat at 50% body mass (η^2^ = 0.40). Large effects were also observed for selected secondary outcomes, including pull-ups with a judogi (η^2^ = 0.39), trunk flexibility (η^2^ = 0.49), and maximal straddle sitting position (η^2^ = 0.37) (all *p* < 0.001). After Benjamini–Hochberg false discovery rate adjustment, most between-group differences remained statistically significant, although secondary outcomes should be interpreted cautiously. In EXP, improvements were observed across multiple outcomes, including 1RM bench press (mean gain $\tilde{x}$ = 5.23 kg), 1RM squat ($\tilde{x}$ = 4.27 kg), pull-ups ($\tilde{x}$ = 1.91 reps), judo-gi hangs (bent arms $\tilde{x}$ = 3.91 s; straight arms $\tilde{x}$ = 4.90 s), and flamingo balance ($\tilde{x}$ = 4.59 s) (all *p* < 0.001). In the EXP group, exploratory correlations suggested that shorter training experience was generally associated with greater conditioning-related improvements, whereas flexibility and balance showed the opposite pattern. **Conclusions**: The GQ intervention package was associated with greater improvements in the motor fitness profile than standard BJJ training. The findings support the potential usefulness of structured circuit-based conditioning as an adjunct to standard BJJ practice, particularly for strength-endurance development. However, the study does not allow the isolated effect of the specific GQ exercise content to be separated from the effect of adding a structured conditioning block, and no direct conclusions can be drawn regarding injury prevention or return-to-play outcomes.

## 1. Introduction

Brazilian Jiu-Jitsu (BJJ) is a combat sport discipline characterised by high technical complexity, substantial intensity, and considerable physical demands [1,2,3]. It requires athletes not only to master grappling techniques, joint locks, and scoring positions but also to maintain excellent functional fitness [4]. Therefore, achieving optimal training outcomes necessitates the development and continuous improvement of several key components, including motor fitness [5] and sport-specific abilities [6,7], exercise capacities [8], and the technical–tactical model [2,9], which collectively form the multidimensional functional profile of the athlete.

In response to the growing competitiveness and specialisation in grappling sports, training programs aimed at providing comprehensive structural and functional preparation for athletes—implemented at various time points within the annual training macrocycle—may gain increasing importance. The sport preparation process continues to evolve, driven strongly by scientific advancements and the adoption of modern training methods [10,11,12,13,14,15]. This trend is particularly evident in well-established Olympic disciplines [16,17]. A key element of this evolution is the search for novel training stimuli designed to maximise the effects of sport preparation, a topic extensively discussed in the literature [18,19]. Such initiatives provide valuable insights both from a scientific and practical standpoint, supporting the effectiveness of the training process.

The currently available BJJ literature indicates that the foundation of training and competitive performance lies in comprehensive motor fitness, with varying degrees of development across specific motor abilities required to meet the demands of training and sport competition [4]. Grappling sports are characterised by a high demand for maximal isometric handgrip strength [20], which appears to be a significant determinant of success in immobilisations, takedowns, throws, and submissions [4]. Research shows that BJJ athletes demonstrate substantially higher levels of grip strength compared with mixed martial arts (MMA) athletes [21] and Muay Thai fighters [22]. Furthermore, athletes are required to possess high levels of absolute strength and explosive power in both upper and lower limbs to gain a decisive advantage over opponents [4]. These capabilities are typically more developed in elite athletes compared with their non-elite counterparts [23]. A consistent body of evidence underscores the critical importance of unique grip strength endurance in BJJ athletes, who clearly outperform competitors from other combat sports in this domain [20,21,22]. More broadly, available studies suggest that global muscular endurance across various body regions is one of the most important determinants of success in BJJ, as athletes must repeatedly perform strength-based actions throughout the course of a match, particularly as its duration increases [4,5,20,21,22,24]. A high level of flexibility has also been identified as an important characteristic of successful athletes, differentiating elite from non-elite performers [25]. Flexibility is especially desirable in the thoracolumbar region and hamstrings, as the optimisation of this structural quality facilitates the adoption of specific positions and supports the acquisition of advanced technical skills [24].

This raises an important question: How can the motor profile of a BJJ athlete be effectively influenced and improved? Existing research on innovative training interventions has shown that a 10-week high-intensity interval training (HIIT) programme applied in Brazilian jiu-jitsu was more effective than standard BJJ training in improving selected elements of the motor profile, such as abdominal strength endurance, BJJ-specific endurance, and movement speed [26]. This innovation concerned the way the training stimulus was operationalised and integrated into BJJ preparation. Similarly, Tropina and colleagues demonstrated that an 8-week dynamic–static exercise program significantly enhanced strength performance in elite BJJ athletes, surpassing the effects of classical BJJ training [19]. Despite these promising findings, the overall effectiveness of such strategies has not yet been comprehensively verified. A review of the literature confirms a substantial research gap in this area, and the scientific community focused on BJJ consistently emphasises the need for further exploration [4].

Therefore, a potentially promising direction would be the design of a training program grounded in the principles of functional training, utilising small, thematic resistance-training circuits [27,28]. Unlike classic HIIT formats, this concept is not defined primarily by the structure of metabolic intervals but by the selection of BJJ-relevant movement tasks and loading patterns implemented in short station-based circuits. It is a pragmatic, BJJ-oriented template for structured conditioning, rather than a claim of superiority over all HIIT-based approaches. Such an approach is intended to improve the motor profile of BJJ athletes, which serves as the foundational determinant for subsequent training and competitive progress. This motor profile underpins the development of further functional components of the training process, including technical–tactical proficiency, sport-specific fitness, and mental preparation. From a broader applied perspective, improved motor fitness may be relevant to athlete health and prehabilitation-oriented training, because strength endurance, flexibility, balance, and neuromuscular control are commonly considered components of physical activity verified through injury surveillance [4]. Moreover, the knowledge gained could be of considerable scientific and practical value—supporting coaches and athletes and contributing to the continued development of the discipline [7,16].

Previous BJJ intervention studies have provided important but still limited evidence. HIIT-based protocols have mainly emphasised metabolic conditioning and repeated high-intensity efforts, whereas dynamic–static exercise interventions have focused more directly on strength and strength-endurance adaptations. The GQ concept differs from these approaches by combining resistance, gymnastic, flexibility, balance, and BJJ-related movement tasks within short station-based circuits. Nevertheless, the present study was not designed to demonstrate superiority over HIIT, dynamic–static training, or any other conditioning method. Rather, it evaluates whether the addition of a structured, BJJ-oriented circuit-conditioning package to standard practice is associated with measurable changes in the motor fitness profile.

Considering the above, the aim of this randomised study was to estimate the effects of the experimental Grappler Quest (GQ) training program on the motor fitness profile of BJJ athletes and to explore whether training experience is associated with the magnitude of the observed changes. Based on previous evidence and practitioner experience, we hypothesised that adding the GQ structured conditioning block to standard BJJ training would be associated with improvements in the motor fitness profile of BJJ athletes, particularly in strength-endurance outcomes.

## 2. Materials and Methods

Ethical approval for this study was granted by the Ethics Committee of the District Medical Chamber in Krakow (No. 226/KBL/OIL/2023). Consistent with the Declaration of Helsinki, participants were fully informed about the purpose and procedures of the research, possible side effects, and their right to withdraw at any time without explanation. All participants provided written informed consent before taking part.

The intervention was training-based and aimed to improve motor fitness and functional readiness (including training-safety–oriented elements), with no clinical endpoints; therefore, clinical trial registration was not applicable [29].

### 2.1. Study Design

An experimental approach with repeated measures and a randomised controlled trial design was applied. The testing procedure was conducted before and after the 8-week intervention period. In the experimental group (EXP), the intervention was integrated into their regular training program, supplemented with thematic GQ circuit-training content. The control group (CON) followed their standard BJJ training program. The experimental stimulus was implemented to allow for a reliable comparison between the training effects achieved under the GQ protocol and those resulting from traditional training methods commonly used in sports clubs.

### 2.2. Participant Characteristics

A total of 44 elite Brazilian jiu-jitsu (BJJ) athletes from Poland participated in the study and were randomly assigned to an experimental group (EXP; *n* = 22) or a control group (CON; *n* = 22). Initially, 48 athletes were recruited; four were excluded because they did not meet the eligibility criteria (injury history and/or current health problems).

Because comparable controlled training interventions in elite BJJ athletes are scarce and recruitment in this highly specialised population is constrained, the sample size was not based on a conventional a priori power calculation for a single confirmatory endpoint. Instead, we conducted an a priori sensitivity analysis to quantify the smallest between-group effect size detectable with the planned ANCOVA model and to support interpretation of both significant and non-significant findings, consistent with methodological recommendations for intervention studies in sport science [30]. The sensitivity analysis was specified for ANCOVA with the post-intervention score as the dependent variable, group (EXP vs. CON) as the fixed factor, and the corresponding baseline score as a covariate [31]. Assuming a two-sided α = 0.05, power (1 − β) = 0.80, and an anticipated baseline–post correlation of r ≈ 0.70, the fixed sample size (*n* = 22 per group) corresponds to a minimum detectable baseline-adjusted standardised between-group difference of approximately Cohen’s d ≈ 0.60. Accordingly, effects smaller than this threshold may have remained undetected, and results should be interpreted with appropriate caution, with emphasis on effect sizes and their uncertainty. Analyses were performed using G*Power v. 3.1.9.6 (Heinrich-Heine-Universität Düsseldorf, Düsseldorf, Germany) [32,33].

Eligibility criteria were defined a priori. Inclusion criteria were (i) ≥4 years of systematic BJJ training; (ii) active competitive participation; (iii) valid medical examination and physician clearance for high-intensity training; (iv) no supplementation during the study period; and (v) willingness to provide written informed consent. Exclusion criteria were (i) current or recent musculoskeletal injury; (ii) history of severe injuries likely to limit training or testing; (iii) cardiovascular or metabolic disorders that could affect training responses; (iv) use of prohibited substances; and (v) absence of written informed consent. These criteria reflected the high physical demands of the intervention and testing procedures.

Participants were 21–31 years old (mean 25.81 ± 3.22 years), with body mass 78.33 ± 10.89 kg, height 177.12 ± 6.20 cm, and BMI 24.92 ± 2.85. Training experience ranged from 4 to 13 years (mean 7.57 ± 2.70 years), with 4–6 training sessions per week depending on the mesocycle. Belt ranks ranged from blue to black (black *n* = 4; brown *n* = 10; purple *n* = 14; blue *n* = 16). Testing was conducted during the preparatory period. None of the athletes reported following restrictive diets. All participants competed regularly at international, national, or local master-class level, and several were medalists at European and Polish championships and other major grappling events. Information on age, training history, and competitive activity was collected via structured interviews with athletes and coaching staff. The study was conducted at the Legion Team Tarnów Sports Club and the Grappling Kraków Sports Club.

Randomisation was stratified by weight category in accordance with International Brazilian Jiu-Jitsu Federation (IBJJF) adult male divisions [34]. The sample included at least four athletes from each of the seven weight categories (64 kg, 70 kg, 76 kg, 82.3 kg, 88.3 kg, 94.3 kg, and 100.5 kg; distribution: *n* = 4, 4, 12, 12, 4, 4, 4, respectively). Within each category, athletes were assigned a unique identification number and allocated to EXP or CON using a computer-generated random sequence (random number generator). The allocation sequence was generated by an individual independent of data collection and training supervision, and assignments were implemented after enrolment to minimise investigator influence. This three-stage sampling procedure (Stages 1–2 purposive; Stage 3 random) produced groups with comparable ages, training experience, and basic structural profiles, with no significant between-group differences (Table 1).

### 2.3. Characteristics of the Experimental Intervention

**Figure 1:**
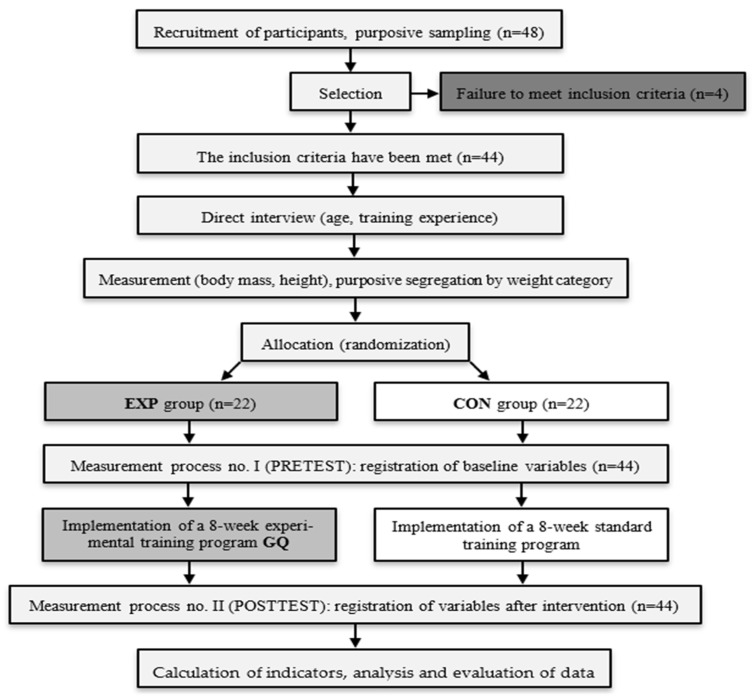
Flowchart of the research intervention.

The intervention was implemented in accordance with the principles of pedagogical experiments [35,36]. The EXP group completed the experimental 8-week GQ training program, which consisted of thematic circuit-training sessions (3 experimental training units per week + 1 sparring unit), while the CON group followed a standard general BJJ training program (3 sessions per week + 1 sparring unit). Training interventions (experimental GQ and standard BJJ training) in both groups were supervised by experienced, certified coaches who were trained by the project authors. Participants received guidance on recommended meals to maintain a balanced diet, appropriate rest, and the importance of maintaining consistent training intensity (adherence to coaching instructions, diligence, and attendance). An attendance log was maintained throughout the study. To determine the profile of changes induced by the GQ training stimulus, two measurement sessions were carried out. The first took place before the start of the mesocycle (baseline diagnostics—pretest). The second session was conducted after completing the 8-week training program (evaluation of effectiveness—posttest). The effect of the GQ program was assessed using intergroup (EXP vs CON) and intragroup comparisons. Only participants with a minimum training attendance of 90% were included in the final analysis (*n* = 44). Figure 1 presents a flowchart of the research intervention.

### 2.4. Characteristics of the Experimental GQ Training Program

The experimental training programme was developed in accordance with functional training guidelines [27] and implemented as short, thematically organised circuit-training sessions [28]. A key design principle was exercise diversity and the use of a wide range of training equipment, with the intention of engaging all major muscle groups through both analytical and integrated movement patterns. The methodological framework of the intervention is summarised in Table 2, and the exercise content is presented in Table 3.

The intervention consisted of three thematically defined circuits performed within each session: (1) a strength-endurance circuit using body mass and external loads (e.g., medicine balls, plates, dumbbells); (2) a functional circuit incorporating BJJ-specific drills performed individually and/or with training aids; and (3) a specialised circuit targeting motor abilities frequently required by BJJ movement patterns. The BJJ-specific drills involved repeated sport-specific actions intended to refine discipline-relevant movement patterns through systematic practice [37].

Each session included three circuits performed in a fixed sequence (I–III), with five stations per circuit (15 stations per session). In Circuit I, each station was completed within a 60 s work interval for 8–15 repetitions, using a controlled tempo of 2-1-2-1. External loads were selected to maintain movement quality and technical correctness, with a practical guideline not exceeding approximately 50% of body mass for loaded exercises (Table 2). In Circuits II and III, each station was performed for time, with 30 s allocated per exercise and a fast-paced execution where appropriate (Table 2). Rest between circuits was standardised at 180 s (Table 2), while transitions between stations were limited to the time required to change position and set up equipment. Progression across the 8-week mesocycle was implemented by increasing repetitions within the prescribed range, increasing task complexity, and/or modestly increasing external load, while maintaining movement quality and the prescribed tempo.

A detailed demonstration of the themed station-based circuits is provided in the Supplementary Material as an instructional video. The video provides a station-by-station walkthrough of the GQ session structure, including exercise setup, execution cues, and work–rest timing. It is intended primarily to support replication of the intervention (and secondarily to illustrate the training concept).

Training sessions were conducted three times per week in the evenings (19:00–20:30), with each session lasting 90 min. Each session began with a 10 min warm-up and concluded with 15 min of specialised stretching targeting body areas where a large range of motion is particularly relevant in BJJ, including the thoracolumbar spine, hamstring complex, posterior knee tendons, and hip joint structures. In addition, a fourth weekly training session was dedicated to sparring practice. The total weekly scheduled training time was 360 min, with the experimental intervention implemented within the three evening sessions.

### 2.5. Characteristics of the Standard Training Program for the CON Group

Before the intervention period, all participants followed their clubs’ standard Brazilian jiu-jitsu (BJJ) training routine. During the study, the CON group continued with standard BJJ training three times per week. A typical session consisted of three parts: (i) a warm-up including general activation and body-conditioning drills; (ii) a main segment comprising BJJ-specific conditioning elements, technical drilling aimed at learning or refining skills, and sparring components (task-specific sparring focused on selected technical–tactical actions and full sparring); and (iii) a final segment including mobility, flexibility, and postural-correction exercises.

When resistance-type work occurred within standard practice, it predominantly involved body-mass exercises and partner-resisted drills rather than a separate, structured circuit-based conditioning block. Partner-based resistance is inherently variable (e.g., partner body mass and interaction dynamics), which limits objective quantification of external load. In addition, gymnastic and plyometric elements were embedded within technical actions typical for BJJ practice (e.g., guard pass with a side flip, gorilla guard pass, guard passes with jumps, ude hishigi juji gatame/flying armbar entry, sankaku jime/triangle jump, and many others, practised in series or individually), often performed in series during drilling. Consequently, conditioning stimuli in the CON group occurred in a more implicit and less standardised manner than in the EXP group, which can complicate precise control over specificity and training dose. Nevertheless, the overall content reflects common training practice in competitive BJJ and has been reported to contribute to sport-specific fitness development [38,39] (see also related qualitative and indirect evidence [4,17]).

A shared element for both groups was one weekly sparring session. To standardise sparring exposure, all sparring sessions followed a predetermined protocol specifying bout duration, number of rounds, and starting position: weeks 1–2, five bouts of 5 min starting from kneeling (ground grappling); weeks 3–4, five bouts of 6 min starting from the ground; weeks 5–6, five bouts of 7 min in full form starting from standing; and weeks 7–8, five bouts of 8 min starting from standing. Coaches were trained in the standardised procedure, and each sparring session was monitored by independent observers to verify protocol adherence. These procedures were intended to keep overall training exposure broadly comparable between groups in terms of planned weekly frequency, session duration, and session structure; however, they describe exposure rather than a comprehensive operational definition of external load.

### 2.6. Measurements of Subjective Exercise Intensity Indices

To assess subjective perceived exertion, the Borg Rating of Perceived Exertion (RPE) scale (6–20 points) [40] was used as a perceptual indicator of internal load (i.e., perceived physiological and psychological strain). Participants were thoroughly instructed on how to interpret the scale, where a value of 6 represents “no exertion” and 20 represents “maximal exertion.” RPE measurements were conducted in both groups during every training session, separately for each part of the session (warm-up, main part, and cool-down). In addition, sparring sessions were also assessed. Measurements covered three thematic activities: standard BJJ training sessions, GQ training, and BJJ sparring. The collected data were averaged and presented in the results section. RPE was used to quantify and compare perceived exertion (internal load) between the EXP and CON groups across session segments and at the session level; however, RPE does not quantify external load (work performed) or training density, and therefore cannot be used to infer equivalence of training dose between conditions.

To estimate subjective internal load, an approach based on the session-RPE method proposed by Foster et al. [41] was applied, consisting of multiplying the RPE value by exercise duration. The session-RPE method may be useful across various exercise modalities, including intermittent and interval-based efforts. Results were expressed in arbitrary units (AU). For the overall comparison of the interventions, mean global RPE values were multiplied by the total training exposure time, whereas for the analysis of the main part of the training sessions, mean RPE values for the main part were multiplied by its duration and the number of completed sessions. Calculations were performed according to the following formula:

Session RPE Load=RPE Session×Duration of the Session in Minutes

### 2.7. Measurement of Objective Indicators of Internal Training Load—Post-Intervention Assessment

To provide an objective and time-efficient characterisation of internal physiological load, heart rate (HR) was assessed during an eight-week monitoring block conducted following completion of the primary 8-week intervention period. Importantly, these HR measurements were not collected during the original intervention period used for the primary pre–post outcome analysis. They were obtained during a standardised reconstruction of the same training protocol and should therefore be interpreted as an approximate characterisation of physiological internal load rather than direct load monitoring from the original trial. The repeated intervention followed the same procedure as the original protocol. HR was recorded using a Polar 610S heart rate monitor (Polar, Finland) at standardised time points: at the end of the warm-up, at the end of the main part of the session, and at the end of the cool-down. During sparring sessions, HR was recorded immediately after the final bout (HR_FINAL_). The collected data were averaged and presented in the Results section. These measurements were used only to describe and approximately compare internal physiological responses between groups during the reconstructed training block; they were not treated as prospective load-monitoring data for the primary intervention.

Subsequently, internal training load was calculated for each part of the training session, sparring sessions, and for the overall training dimension using recently developed sport-specific formulas for HR index and training load, methodologically proposed by Ambroży and Rydzik [42]:

$$Index\;HR=\frac{\left(HRs-25\right)}{25}$$

where

HR—heart rate session (i.e., warm-up, main part, final part, and sparring sessions).

(1) Training Load=Index HR×Duration of the Session in Minutes

where

Duration of the Session—duration of each individual session (i.e., warm-up, main part, final part, and sparring sessions).

Results were expressed in arbitrary units (AUs).

### 2.8. Motor Fitness Measurements

Before each testing session, both groups participated in a 15 min warm-up consisting of exercises preparing the body for physical effort. A sequence of conditioning exercises was used, including static and dynamic movements of the arms, trunk, abdomen, back, legs, and head. Subsequently, the examiner demonstrated each test according to the standardised procedure and provided instructions and clarifications. All measurements were conducted at dedicated testing stations in the BJJ training hall where regular training sessions take place. Only one group was present in the testing area at any given time.

Motor fitness, in the context of selected motor abilities, was assessed using standardised tests specific to BJJ (*n* = 3) and non-specific tests (*n* = 13). The selection of tests was based on an in-depth review of the relevant literature and many years of athlete–coach experience (tests subjectively considered most useful for assessing the physical demands placed on BJJ athletes, with emphasis on the function of muscle groups responsible for resisting and generating external and internal force). The testing procedure included the following assessments:

Strength assessments
  1. Static handgrip strength measurement (assessment: maximal isometric handgrip strength). Standing with feet slightly apart, the participant held the dynamometer firmly in the fingers, with the arm along the torso, ensuring the device did not contact the body. A brief maximal grip effort was performed while the other arm remained relaxed. The best value from two maximal attempts with the dominant hand (HGSmax) was recorded using a dynamometer (MG 4800, Charder, Taichung, Taiwan), accurate to 1 kg [43].
  2. Barbell bench press, 1 repetition maximum (1-RM; assessment: maximal dynamic strength of the upper body, i.e., pectoralis major, triceps brachii, anterior deltoids, trunk stabilisers).
  3. Barbell back squat, 1 repetition maximum (1-RM; assessment: maximal dynamic strength of the lower body, i.e., quadriceps, gluteals, hamstrings, trunk stabilisers).

For both bench press and squat, participants completed a warm-up set of 5 repetitions at ~50% of estimated 1-RM, followed by 3 repetitions at 70% of estimated 1-RM, with 2 min intervals. Athletes then attempted their 1-RM in at least three and up to five trials, with 3–5 min rest intervals, following the recommendations of Brown and Weir [44].

Strength endurance assessments
  4. Pull-ups on a judogi (assessment: strength endurance of the arm and back muscles). Hanging from a judogi placed over a pull-up bar, the participant performed the maximum number of pull-ups (from full arm extension to maximal elbow flexion). The test was performed once [45]. This test is specific to Judo and BJJ.
  5. Bent-arm hang on a judogi (assessment: functional strength/isometric muscle endurance). The test involves hanging from a kimono suspended on a bar, with arms maximally flexed at the elbows, maintaining the position for as long as possible. The stopwatch starts when the participant begins hanging independently. The time measurement continues until the elbows extend beyond 90 degrees. The test is performed once. Hanging time is recorded with an accuracy of 0.1 s [45]. This trial is specific to Judo and BJJ.
  6. Straight-arm hang on a judogi (assessment: functional strength/isometric muscle endurance). The participant maintained a dead-hang position with elbows extended for as long as possible. The stopwatch stopped when the grip failed. Recorded with 0.1-s precision. Specific to Judo and BJJ.
  7. Sit-ups (assessment: strength endurance of the abdominal muscles). The participant lay on a mat with feet 30 cm apart and knees bent at a 90-degree angle. Hands were clasped behind the neck, and a partner held the participant’s feet. At the start signal, the participant sat up, touched the knees with elbows, and returned to the starting position. The duration was 30 s, and the result was the number of repetitions completed [43].
  8. Back extensions (assessment: strength endurance of back extensor muscles). The participant lies prone on a mat with hands clasped behind the neck. Upon signal, they lift their torso and legs off the ground, arching the back by engaging the back muscles, then return to the starting position as quickly as possible. This movement is repeated as many times as possible within 30 s. The examiner counts the number of repetitions performed. For example, 18 correctly executed repetitions yield a score of 18. The test is performed once [43].
  9. Bench press at 50% body mass (assessment: upper-body strength endurance). From a supine position on a flat bench, the participant performed the maximum number of repetitions using a load equal to 50% of their body mass. One trial was performed [22,46].
  10. Back squat at 50% body mass (assessment: lower-body strength endurance). With a barbell positioned across the shoulders, participants performed the maximum number of repetitions with 50% body mass. One trial was performed [22,46].
Speed and power assessments
  11. 10 × 5 m shuttle run (assessment: speed, agility, and running endurance). Participants sprinted between two lines spaced 5 m apart, crossing each line with both feet, repeated 10 times. Time recorded to the nearest 0.1 s [43].
  12. Standing long jump (assessment: explosive lower-limb strength). The participant stands with feet slightly apart behind the start line, bends the knees, swings the arms backwards, and then jumps forward as far as possible, landing on both feet while maintaining an upright position. The test is performed twice, and the longest jump is recorded. The distance is measured to the nearest heel mark to the nearest centimetre. A measuring tape, a firm surface, and two connected gymnastics mats are used [43].
Coordination assessments
  13. Flamingo balance test (assessment: static balance). The participant stands on a bar measuring 50 cm in length,4 cm in height, and 3 cm in width. The participant holds the free leg bent at the knee, grasping the foot. The goal is to maintain balance for as long as possible. The measurement ends when the participant loses balance, releases the leg, or touches the ground. A preparatory trial is allowed before the measurement. Time is recorded with an accuracy of 0.01 s [43]. Test according to Żak’s modification [47].
  14. Simple reaction time to visual stimulus. The test is conducted using a computer keyboard. Active keys: “Enter” on the right for the right hand and “Shift” on the left for the left hand. The participant rests their hand on the table next to the keyboard, with the thumb or index finger on the active key. When a bright square appears in the centre of the screen, the participant must press the key as quickly as possible. The test consists of 11 irregularly timed stimuli. The faster the reaction, the better the score. The examiner demonstrates the task, provides instructions and explanations, then the participant performs a practice trial with 5 stimuli before proceeding to the main test with 11 stimuli [48].
Flexibility assessments
  15. Forward trunk flexion, sit-and-reach (assessment: trunk flexibility). The test is conducted as a seated reach movement, with the range measured in cm beyond foot level. While sitting, the participant extends their arms forward as far as possible, reaching forward, with legs extended. A ruler slides along the surface of a box with a pre-prepared scale. The better of two attempts is recorded. If the participant reaches 10 cm beyond the toes, the score of 10 is awarded. The box measures 40 cm in length, 45 cm in width, and 35 cm in height, with a scaled top extending 25 cm beyond its front edge, serving as a footrest. The surface features a 65 cm-long scale, with a ruler aligned perpendicularly to the box’s axis for forward reach measurement [43].
  16. Maximal straddle sitting position (assessment: hip joint flexibility). Participants performed a maximal straddle sit facing a wall, with feet touching it and knees extended. The distance from the pubic symphysis to the wall was measured in centimetres [49].

Timing of test sessions: each group completed four measurement sessions, each lasting 120 min. The minimum recovery period between sessions was 22 h. Between individual tests, effective rest intervals of at least 20 min were observed.

1. Session 1—Strength assessment. Order: bench press 1-RM → squat 1-RM → handgrip dynamometry. Recovery before next session: 46 h.
2. Session 2—Strength endurance assessment, Part 1. Tests: bench press 50% BM → squat 50% BM → sit-ups → bent-arm hang on judogi. Recovery before next session: 22 h.
3. Session 3—Coordination and speed assessment. Tests: flamingo balance → reaction time → standing long jump → shuttle run. Recovery before next session: 22 h.
4. Session 4—Strength endurance assessment, Part 2. Tests: pull-ups → back extensions → straight-arm hang on judogi.

### 2.9. Motor Indicators

The results obtained during direct measurements were used for indirect estimation of relative strength indicators (RS), including relative static handgrip strength (RS_SH_), relative strength in the 1RM bench press (RS_BP_), relative strength in the 1RM squat (RS_S_), relative strength in pull-ups on a judogi (RS_PU_), and relative hanging endurance (RS_H1_, RS_H2_) [46]—calculated as the ratio of the measured variable (maximum local static hand strength [kg], maximal eccentric–concentric strength in the bench press and squat [kg], maximal number of pull-up repetitions [reps], maximum hanging time [s]) to the participant’s body mass according to the following formula:

$$RS=\frac{test\;result\;[kG;kg;reps;s]}{body\;mass\;[kg]}$$

RS is a strength indicator representing the ratio of muscle-generated force to total body mass. This measure provides an objective and accurate representation of actual muscle strength, which is particularly important in weight-class-restricted sports [50], including BJJ [34].

### 2.10. Statistical Analysis

Statistical analysis was performed using PQStat v1.8.6 (PQStat Software, Poznań, Poland) and Statistica 13.3 (StatSoft, Kraków, Poland). Between-group differences were primarily assessed using analysis of covariance (ANCOVA), with the post-intervention value as the dependent variable, group (EXP vs. CON) as the fixed factor and the corresponding baseline value as a covariate. Separate ANCOVA models were fitted for each outcome. Results are reported as adjusted means with 95% confidence intervals (95% CI), together with F statistics, *p*-values, and partial eta-squared (ηp^2^) as the measure of effect size. Effect sizes were interpreted using conventional thresholds (ηp^2^ ≥ 0.01 small, ≥0.06 moderate, ≥0.14 large). Given the number of outcomes analysed, *p*-values were interpreted cautiously and alongside effect sizes and their uncertainty (95% CIs), rather than as the sole basis for inference. A two-sided *p*-value < 0.05 was used as the nominal threshold for statistical significance. In this randomised trial, two primary outcomes were prespecified to reflect the main hypothesis regarding strength endurance: the 50% body-mass bench press and the 50% body-mass squat. These outcomes were selected because strength endurance is repeatedly identified as a key physical determinant in BJJ, where athletes must perform repeated forceful pushing, pulling, gripping, bridging, lifting, and positional-control actions over several minutes of intermittent combat. The bench press and squat performed with 50% body mass were chosen as standardised, reproducible indicators of upper- and lower-body strength endurance that could be safely applied across weight categories and compared between groups. They were also directly aligned with the main expected adaptation of the GQ intervention, which emphasised repeated submaximal resistance efforts within circuit-based work. All other outcomes were treated as secondary/exploratory. To address multiplicity arising from testing multiple outcomes, *p*-values from the ANCOVA models were additionally adjusted using the Benjamini–Hochberg false discovery rate (FDR) procedure (q = 0.05), and both unadjusted *p*-values and FDR-adjusted q-values are reported. The assumption of Homogeneity of Regression Slopes (HRS) for ANCOVA was tested by examining the interaction between the group factor (EXP vs. CON) and baseline scores, and found to be non-significant (*p* > 0.05), confirming the validity of the ANCOVA results. Within-group pre–post changes were explored using a paired *t*-test or, when distributional assumptions were not met, the Wilcoxon signed-rank test. Baseline group comparability was summarised descriptively; where baseline hypothesis tests were reported, independent-samples *t*-tests or Mann–Whitney U tests were applied depending on distributional assumptions (placed for illustrative purposes in the Appendix A). Standardised mean differences were additionally expressed as Cohen’s d (0.20 small, 0.50 moderate, 0.80 large). Exploratory analyses of associations between training experience and training-related changes were conducted using Spearman’s rank correlation (rs). Correlation magnitudes were interpreted as follows: 0.00–0.19 very weak, 0.20–0.29 weak, 0.30–0.49 moderate, 0.50–0.79 strong, and >0.80 very strong. The selection of parametric versus non-parametric tests was guided by the Shapiro–Wilk test for normality and Levene’s test for homogeneity of variance [51].

## 3. Results

### 3.1. Baseline Characteristics

In the baseline between-group assessment (pre-test), the research groups (EXP vs. CON) demonstrated a comparable motor profile in terms of conditioning and coordination abilities (*p* > 0.05). The only exception was simple reaction time (a component of coordination abilities), for which a significant difference was observed in favour of the EXP group (*p* = 0.003). The CON group showed a non-significant advantage in strength, strength-endurance, and explosive power, whereas the EXP group demonstrated a non-significant advantage in speed, coordination, and flexibility characteristics (Appendix A—Table A1).

### 3.2. Post-Intervention Results: ANCOVA Analysis

The ANCOVA models (post-intervention value as the dependent variable, group as the factor, and the corresponding baseline value as a covariate) indicated between-group differences favouring the EXP group across most outcomes. Specifically, the EXP group showed more favourable adjusted post-intervention values in 18 of the 22 motor variables (nominal *p* < 0.05). The largest effects were observed for strength-endurance outcomes in the bench press at 50% body mass (ηp^2^ = 0.52) and squat at 50% body mass (ηp^2^ = 0.40) (prespecified primary outcomes), as well as (secondary outcomes) pull-ups performed in a judo gi (ηp^2^ = 0.39) and flexibility measures, including trunk flexibility (ηp^2^ = 0.49) and maximal straddle sitting position (ηp^2^ = 0.37) (all nominal *p* < 0.001) (Table 4).

After controlling for multiple comparisons using the Benjamini–Hochberg FDR procedure, 18 of the 22 outcomes remained statistically significant (q < 0.05); back extensions, sit-ups, standing long jump, and simple reaction time were not significant after FDR adjustment (Appendix A—Table A2). Accordingly, interpretation of secondary outcomes should be considered supportive and more uncertain than that of the prespecified primary outcomes.

### 3.3. Intra-Group Analysis: Pre- and Post-Intervention Comparisons

Table 5 presents the results and direction of changes in the components of the motor profile that occurred in the studied groups following the experimental intervention (pretest vs. posttest), as well as the degree of their within-group variability.

The comparative analysis revealed significant differences (pretest vs. posttest, *p* < 0.05) indicating a more favourable level across most tested motor variables within the EXP group. Exceptions included running speed in the 10 × 5 m shuttle run and simple reaction time (*p* > 0.05). The most pronounced improvements were observed in, among others, the 1RM bench press (median increase $\tilde{x}$ = 5.23 kg), 1RM squat ($\tilde{x}$ = 4.27 kg), pull-ups ($\tilde{x}$ = 1.91 repetitions), bent-arm and straight-arm hangs on a judogi (bent arms: $\tilde{x}$ = 3.91 s; straight arms: $\tilde{x}$ = 4.90 s), and static balance in the flamingo test ($\tilde{x}$ = 4.59 s) (all *p* < 0.001).

In the CON group, after 8 weeks of planned and completed standard BJJ training, a significant improvement was observed in sit-ups ($\tilde{x}$ = 0.59 repetitions), as well as in 1RM bench press ($\tilde{x}$ = 1.27 kg) and hanging endurance on a judogi (bent arms: $\tilde{x}$ = 0.87 s; straight arms: $\tilde{x}$ = 1.15 s), both in absolute and relative (RS) metrics (*p* < 0.05). For the remaining variables, no significant within-group changes were noted (*p* > 0.05) (Table 5).

### 3.4. Correlation Analysis Between Training Experience and Training Effects in the EXP Group

Table 6 presents exploratory correlation coefficients between training experience (TE) and the magnitude of training effects obtained from the GQ experimental intervention within the EXP group.

Clear associations were identified (*p* < 0.001) between TE and the straight-arm hang test, as well as its relative measure, with correlations of very high strength and a negative direction (r_s_ = −0.83 and –0.82). For several variables (handgrip dynamometry, 1RM bench press + RS, pull-ups, bent-arm hang on a judogi + RS, standing long jump, sit-ups, 50% 1RM bench press and squat), the same trend was observed, with strong negative correlations (r_s_ ranging from −0.68 to −0.79). Moderate correlations were noted for trunk extension and 1RM squat with RS (r_s_ = −0.47 and −0.45; all *p* < 0.05).

Interestingly, in the case of static balance and trunk flexibility, opposite positive and strong relationships were found (r_s_ = 0.53 and 0.50). For the maximal straddle position, where a lower score indicated better performance, co-occurrence with a moderate (borderline strong) negative correlation was observed (r_s_ = −0.48) (all *p* < 0.05).

### 3.5. Between-Group Comparison of Subjective Internal Load (RPE)

The averaged values of subjective perceived exertion (RPE) recorded during the 8-week intervention were as follows: in the warm-up phase, EXP = 10.27 ± 0.77 and CON = 10.36 ± 0.66 (*p* = 0.675; d = 0.13), indicating moderate effort. In the main phase, values of EXP = 13.91 ± 1.66 versus CON = 13.32 ± 2.03 were observed (*p* = 0.297; d = 0.32; very intense effort), whereas in the cool-down phase the values were EXP = 8.77 ± 0.97 versus CON = 8.41 ± 0.50 (*p* = 0.127; d = 0.49; light effort). During the sparring sessions, which were conducted jointly for both groups, RPE values were EXP = 16.23 ± 2.20 and CON = 16.32 ± 2.32 (*p* = 0.895; d = 0.04; very hard effort). The averaged data for total training activity over the intervention period (training sessions plus sparring) were EXP = 12.3 ± 3.31 versus CON = 12.1 ± 3.4 (*p* = 0.703; d = 0.06). No statistically significant differences between groups were observed in any phase of the training, indicating comparable perceived exertion in the EXP and CON groups throughout the intervention period.

With identical intervention durations in both groups (2880 min), the estimated total subjective internal load amounted to 35,424 AU in the EXP group and 34,848 AU in the CON group. The between-group difference was 576 AU (1.65%), indicating a very similar overall level of subjectively perceived training load. Importantly, within the main part of the training sessions, the total load reached 21,699.60 AU in the EXP group and 20,779.20 AU in the CON group. The difference amounted to 920.40 AU (4.43%), indicating comparable, although slightly higher, internal load during the main training component in the EXP group.

### 3.6. Between-Group Comparison of Heart-Rate Responses During the Reconstructed Post-Intervention Training Block

Mean values of the internal-load component, i.e., HR, recorded during the reconstructed 8-week intervention were as follows: during the warm-up phase, EXP = 120.33 ± 7.48 bpm and CON = 120.67 ± 6.88 bpm (*p* = 0.876; d = 0.05). In the main phase, values were EXP = 170.88 ± 5.27 bpm versus CON = 169.12 ± 5.80 bpm (*p* = 0.298; d = 0.32), whereas in the cool-down phase, values were EXP = 90.24 ± 6.50 bpm versus CON = 89.55 ± 6.90 bpm (*p* = 0.734; d = 0.10). During sparring sessions, conducted jointly for both groups, HR parameters were EXP = 180.44 ± 6.18 bpm and CON = 179.18 ± 5.90 bpm (*p* = 0.493; d = 0.21). Mean data for total training activity during the 8-week reconstructed intervention period were EXP = 140.47 ± 37.66 compared with CON = 139.63 ± 37.15 (*p* = 0.748; d = 0.02). No statistically significant between-group differences were observed in any part of the reconstructed training or in the averaged global dimension.

The recorded internal training load during the warm-up phase was EXP = 915.17 AU versus CON = 918.43 AU, with a difference of 0.36%. In the cool-down phase, values were EXP = 939.46 AU versus CON = 929.52 AU (1.07%). During sparring sessions, conducted jointly for both groups, loads were EXP = 4476.67 AU and CON = 4440.38 AU (0.82%). Importantly, despite the different structure, specificity, and exercise content, the main phase showed values of EXP = 9102.91 AU versus CON = 8993.09 AU (1.22%), whereas the global internal loads for the total training activity during the 8-week reconstructed intervention period were EXP = 13,302.14 AU compared with CON = 13,205.38 AU. The difference was 96.76 AU (0.73%), suggesting similar reconstructed physiological internal load between groups. Because these HR data were obtained during a post-intervention reconstruction of the protocol, they should be interpreted as supportive descriptive data rather than as direct evidence of equivalent physiological load during the original intervention. Accordingly, HR-based results should not be presented as equivalent to prospective monitoring performed during the primary trial.

## 4. Discussion

In contemporary periodisation, unconventional stimuli tailored to the effort profile and key performance determinants of a given discipline are widely implemented, supporting the overall training process [52,53,54]. To the best of the authors’ knowledge, the present study is one of the few reports evaluating the effectiveness of an innovative 8-week training program on the motor performance of competitive BJJ athletes—and certainly the first to approach the problem using such a multidimensional methodology. The key findings of this intervention indicate that the Grappler Quest (GQ) program was associated with improvements in the motor profile of the EXP group, including outcomes related to strength, strength endurance, motor coordination, and flexibility. In the between-group ANCOVA analysis (Table 4) and within-group pre–post comparisons (Table 5), statistically supported differences were observed (nominal *p* < 0.05), suggesting a broad pattern of motor fitness improvements in the EXP group. After controlling for multiple comparisons using the Benjamini–Hochberg FDR procedure (Appendix A Table A2), the overall pattern of between-group results remained largely unchanged. Another important finding relates to training experience within the EXP group. Exploratory correlation analyses suggested that, for most motor abilities assessed, shorter training experience was associated with larger training-related changes. Balance and flexibility were exceptions to this pattern, showing opposite associations (Table 6). Importantly, interpretation should prioritise the prespecified primary outcomes, whereas secondary outcomes should be treated as supportive.

Although the present intervention was performance oriented rather than therapeutic, the observed improvements may still be relevant to applied clinical sports medicine in grappling athletes. It is well established that comprehensive motor preparation, combined with prehabilitation and neuromuscular training, represents one of the most effective methods for reducing the risk of sports injuries [55,56,57]. Appropriate motor preparation should not only enable an athlete to function, first, stronger, faster, and with greater endurance, but also ensure safe training participation with a reduced risk of injury. This is particularly important in combat sports, where injuries are difficult to eliminate completely; therefore, efforts should focus on minimising their occurrence and delaying their onset as long as possible. In our opinion, the GQ approach may have contributed to improving the so-called weak links in the motor fitness profile of athletes in the EXP group, which may potentially be associated with reduced injury susceptibility, as deficits in motor abilities are considered important intrinsic risk factors for injury. When rationally programmed, with appropriate moderation of training volume and intensity, GQ may also be considered as a supportive element in the return-to-training process following injury or a prolonged training break [58]. Brazilian jiu-jitsu is associated with a substantial burden of musculoskeletal injuries, particularly during sparring, and fatigue-related decrements in neuromuscular control and movement quality are commonly discussed mechanisms linking conditioning status to injury risk. From a preventive perspective, greater strength endurance, improved flexibility, and better postural control may help athletes tolerate repeated high-force isometric and dynamic actions and potentially reduce susceptibility to technique breakdown under fatigue. In addition, structured circuit-based conditioning may align with late-stage return-to-play frameworks by providing a progressive bridge from general physical preparation toward sport-specific work capacity. The proposed clinical implications are consistent with the direction of the available literature [55,56,57,58] and were formulated indirectly on the basis of qualitative reasoning, which is considered equally important as quantitative inference [17]. In the present study, relationships between improvements in motor performance induced by GQ and the occurrence, prevention, or treatment of injuries were not directly assessed. These interpretations should be treated as mechanistic and hypothesis-generating, not as evidence that GQ reduces injury risk. Future studies should combine motor-performance testing with prospective injury surveillance and return-to-training outcomes to verify these potential applications.

It is already known that the GQ program effectively influenced the structural profile of BJJ athletes [59]. Training and competitive activity in BJJ is also largely determined by a high level of motor performance [4], which constitutes the foundation of athletic development. Technical–tactical skills, sport-specific fitness, and mental preparedness are all built upon this base [5]. In line with the principle of continually advancing the discipline, this encourages the search for optimal strategies for progression. Therefore, it was assumed that the GQ intervention—a compilation of resistance, gymnastics, and flexibility exercises combined with BJJ-specific elements implemented in small training circuits—could contribute to improvements in the motor profile of the studied athletes.

Indeed, the within-group analysis in the EXP group indicated changes in the motor profile across multiple outcomes. The post-test results indicate that the athletes improved their maximal dynamic strength (1RM bench press, 1RM squat), absolute isometric static strength (handgrip dynamometry), strength endurance (pull-ups, bent-arm and straight-arm hangs on a judogi, 50% BM bench press and squat repetitions, sit-ups and trunk bends), flexibility (forward trunk flexion, maximal straddle position), and static balance—a component of coordinative motor abilities (CMA) [50]. Improvements were also noted in relative terms (RS). This is both interesting and important for BJJ, as the discipline is structured around weight categories [34], and the relative dimension of strength and strength endurance (per kilogram of body mass) plays a significant role in training and competitive performance [5]. The between-group comparison of adjusted means (ANCOVA) indicated differences favouring the EXP group relative to the CON group across several motor outcomes. The magnitude of these differences ranged from moderate to large (η^2^ = 0.19–0.52).

Similar directions of training effects have been observed in numerous studies—primarily within established Olympic combat sports—examining the effectiveness of experimental programs combining diverse exercise content and varied modes of implementation [60,61,62,63,64,65,66,67,68,69,70,71]. Focusing on strength abilities in judo, Franchini et al. demonstrated that 8 weeks of linear and undulating strength training produced significant improvements in handgrip strength (average increases of 4.6% and 6.1% for the right and left hand, respectively), 1RM bench press (11.6%), and 1RM squat (7.1%) [60]. Similar conclusions were reported in another study involving judo athletes, emphasising that the analysed variables exhibited greater gains in strength endurance adaptations than in maximal strength in a single exercise [61]. This pattern resembles the trends observed in the present study, considering the effect sizes recorded. Interestingly, a significant improvement in strength abilities (squat: +16%; bench press: +13.7%) was also observed among female judo athletes following an 8-week intervention [62]. Further evidence comes from a 4-week contrast training protocol conducted among boxers, which improved 1RM bench press by an average of 6.4% [63]. Continuing with findings in boxing, two studies demonstrated increases in muscular strength and power (bench press, squat, jump squat), highlighting the importance of combining strength training and plyometrics to enhance performance in elite athletes [64,65]. Karate practitioners participating in 7 weeks of plyometric or cluster training recorded significant improvements in 1RM squat and explosive power in the countermovement jump (CMJ). Moreover, both protocols appeared equally effective in optimising motor performance [66]. A divergent outcome was reported by Özbay et al. in wrestlers, where 30 days of CrossFit training did not produce improvements in the analysed variables (bench press, squat, grip strength), although motor performance was maintained [67]. Considering the above findings, it can be stated that the GQ program was effective in generating moderate increases in strength among the studied athletes. They achieved improvements of 5.4% in grip strength, 5.9% in bench press, 3.9% in squat, and 0.7% in explosive lower-limb power measured by the standing long jump.

With regard to strength endurance, judokas participating in an 8-week undulating training program demonstrated improved training effects in pull-ups on the judogi (increasing on average from 14 to 17 repetitions, corresponding to a 21.4% improvement), whereas those exposed to linear training progressed in the bent-arm hang on the judogi by 28.1% (from 32 to 41 s). They also increased their total lifted load at 70% 1RM in the bench press (15.1%) and squat (9.6%). However, no significant improvements were observed in the number of repetitions performed at 70% 1RM in the bench press and squat cycles [60]. Ambroży et al. reported that 8 weeks of CrossFit training improved general and specific physical fitness in kickboxers, with distinctly noticeable enhancements in strength endurance measured by pull-ups (12.9%) and sit-ups (5.8%) [68]. In another study on sport ju-jitsu athletes subjected to an experimental training program, significant improvements were noted in pull-ups (24%), sit-ups (13.7%), barbell bench press (23.5%), and squat (30.7%), each performed with a load equal to the athlete’s body mass [69]. Only two studies were found addressing unconventional training stimuli in BJJ athletes [19,26]. Ribeiro et al. reported that 10 weeks of HIIT (High-Intensity Interval Training) significantly improved abdominal endurance (average improvement of 29.8%), as well as BJJ-specific speed (20.7%) and endurance (18.9%) [26]. Tropin et al. found that 8 weeks of static–dynamic exercise training significantly improved strength and strength-endurance performance in BJJ athletes, with increases ranging from 4.5% to 22% [19]. Our hypothesis assumed that the greatest sensitivity of the participants to the GQ intervention would occur precisely in this domain. The results confirm these assumptions. Improvements were observed in every strength-endurance variable: upper back and shoulder girdle muscles (pull-ups: +14.3%), chest and arm muscles (bench press at 50% BM: +5.0%), lower limb muscles (squat at 50% BM: +6.2%), abdominal muscles (sit-ups: +3.2%), back muscles (trunk extensions: +1.7%), isometric upper-limb strength in hanging tasks (bent-arm hang: +9.7%; straight-arm hang: +9.5%). When compared with the aforementioned studies, this points to a strong and moderate optimisation of the analysed manifestations of strength endurance. This is highly relevant given the specific demands of BJJ, where a high level of these capabilities is crucial—particularly the sport-specific and unique isometric endurance of the upper limbs required for prolonged grip retention on the gi [4,5].

Nagl’s research demonstrated improvements in both static balance (by 66.4%) and dynamic balance (25%) in female karate athletes performing the kata Gankaku after 8 weeks of resistance exercises using a Swiss ball. These effects were attributed to enhanced strength of the abdominal and back muscles (the trunk), which serve as the primary centre for distributing movement during technique execution, leading to a higher level of trunk stability and, consequently, whole-body balance [70]. A significant increase in dynamic balance was also observed following an 8-week plyometric and Pilates intervention among karate athletes, with plyometrics being the more effective method [71]. Conversely, an experimental intervention did not improve static balance among sport ju-jitsu athletes [69]. Our findings indicate high sensitivity and trainability of static balance within the GQ protocol (improvement of 32.3%). The training included balance-oriented content (e.g., exercises on a gymnastic ball, drills on the heavy bag, gymnastics—see methodology section). This may also be attributed to improved muscle contraction capacity in the lower limbs or changes in proprioception and neuromuscular control, as these are essential for enhancing balance [72]. From a practical standpoint, a high level of this ability appears to be an important component in BJJ—particularly for maintaining or rapidly regaining balance after being destabilised (this applies mainly to the standing phase of combat—throws and takedowns—but also to defending sweeps in ne-waza). Unfortunately, the lack of available research within combat sports on the progression of this variable prevented a broader discussion of this aspect. This highlights the need for further research in this direction.

For flexibility, Eken et al. demonstrated that implementing a warm-up protocol based on static stretching exercises was an effective method for developing flexibility in judokas [73]. A similar direction was observed in our study, although static stretching exercises were included in the final part of the training session (see methodology section). Likewise, another study on judokas showed that increases in flexibility can also be achieved through resistance-training programmes [74]. Training with a Swiss ball among karate athletes also generated improvements in flexibility characteristics [70]. The flexibility improvements observed in our study (an increase of 9.8% in maximum trunk forward bend and 6.4% in maximum straddle width) may be related to the fact that resistance exercises performed through a full range of motion, engaging both agonist and antagonist muscle groups (as implemented in GQ), can more effectively enhance flexibility [75], because from a neuroanatomical perspective, such a stimulus represents an effective form of proprioceptive neuromuscular stretching [73]. Alongside a well-rounded motor profile, flexibility is an essential component in BJJ. A high level of flexibility facilitates athletes in adopting specialised combat positions and in learning demanding technical skills during training [24].

It should also be noted that athletes in the CON group demonstrated within-group progress, with significant improvements in selected motor variables. A significant improvement was observed in seven out of 22 parameters describing their motor profile (1RM bench press + RS, straight-arm and bent-arm hangs on the judogi + RS, sit-ups; all *p* < 0.05). This illustrates that the structure of BJJ training and the complexity of conducting sport combat in this discipline determine the development of athletes’ motor abilities. However, the number of meaningful progressions was lower, and their magnitude was smaller than in the EXP group. This, in turn, suggests that traditional training methods, although beneficial, may not be sufficiently effective at every stage of periodisation for improving key motor abilities that determine optimal training and competition readiness [20].

The between-group comparison indicated differences between groups for most variables (Table 4). Notably, the EXP group showed more favourable final levels in 21 out of 22 variables, thereby reversing the trend observed before the intervention when compared to the CON group (only five out of 22 were more favourable in the CON group). It is worth emphasising that future studies should consider different exposure periods, involving longer application times of the discussed or similar stimuli, to verify the potential for generating even stronger training adaptations with more pronounced changes. Importantly, the direct between-group comparison allows for the identification of directional trends in improvement of the motor profile (eliminating existing differences or expanding them, as well as shifting the level to a more favourable one compared to the pre-test) in the EXP group, when compared to the control group. The more favourable adjusted outcomes in the EXP group indicate that, under the present design, adding the structured GQ package to standard practice was associated with greater motor-fitness gains than standard practice alone.

The applied combination of training stimuli was associated with broad changes in the EXP group within the GQ programme. This level of improvement was not observed in the CON group, which followed a standard BJJ training regimen. The results of subjective ratings of perceived exertion (RPE) enabled comparisons between the study groups. Similar RPE values were observed both across individual components of the training sessions and throughout the entire intervention period. Subsequently, the calculated estimated subjective internal load based on session-RPE [41] demonstrated the same trend. Likewise, the recorded HR parameters and the calculated internal load values [42] obtained during the reconstructed intervention, for each part of the training session, sparring sessions, and the global mesocycle dimension, revealed a consistent pattern. This indicates a standardised training exposure between the groups. Importantly, these findings are consistent with previous reports from combat sports, including Brazilian jiu-jitsu, regarding RPE responses recorded during sparring and training sessions [3,40,76,77]. This suggests that the observed differences are more likely attributable to the structured and specific nature of the GQ training program rather than to variations in total training load or exercise content. Accordingly, it is recommended that GQ be incorporated into the broader periodisation of the training process, that is, implemented within the “optimal window” of the preparatory period within the macrocycle. GQ can be integrated into BJJ training cycles as a complementary and function-oriented method. The protocol may be particularly useful during the general preparatory phase or the immediate pre-competition phase, where improvement of motor readiness is most beneficial [28,40]. Coaches may adjust the frequency and intensity of the programme to technical and tactical training, thereby providing athletes with improved motor adaptation. Such interventions may also be highly relevant for enhancing sport-specific fitness [68]. Periodising GQ alongside regular sparring and technical–tactical training may optimise competitive performance, highlighting its practical value within the broad framework of the training process.

The present findings indicate that a structured and planned conditioning stimulus can provide an additional adaptive challenge within BJJ preparation. However, this should not be interpreted as evidence that traditional BJJ training is ineffective. Rather, standard BJJ practice and structured conditioning may serve complementary roles depending on the phase of periodisation and the targeted motor qualities.

However, these measures do not fully characterise the external training stimulus, movement specificity, training density, or mechanical loading pattern. Therefore, the present findings should not be interpreted as proof that the specific exercise content of GQ alone was responsible for the observed adaptations. Rather, the results support the effectiveness of the overall intervention package, which combined structured circuit organisation, progressive conditioning content, and BJJ-oriented movement tasks. This distinction is important because the control group followed routine practice, whereas the experimental group received a more standardised conditioning block. Accordingly, future research should compare GQ with other matched conditioning formats to determine whether its specific exercise selection provides benefits beyond structured conditioning itself. The practical significance of the observed changes should be interpreted in the context of BJJ-specific demands rather than only by statistical significance. At present, no well-established minimal practically important change thresholds are available for most motor tests used in competitive BJJ athletes. Therefore, the practical relevance of the present findings was inferred from the magnitude and direction of change, the between-group adjusted differences, and the functional demands of grappling. Improvements in bench press and squat endurance at 50% body mass may be practically meaningful because BJJ requires repeated submaximal force production during positional control, escapes, guard passing, takedown defence, and prolonged isometric–dynamic exchanges [4,20,21,22,24]. Importantly, the improvement in strength endurance encompassed both upper- and lower-body muscle groups, which may contribute to effectiveness in the multifaceted demands of BJJ performance. This may be particularly relevant during contests between two functionally comparable opponents, where the match extends over the full regulation time. Similarly, improvements in judogi pull-ups and hanging tasks may have direct relevance to gi-based gripping actions. It is well established that BJJ places a unique demand on the ability to sustain prolonged gripping actions on an opponent’s gi or body. Flexibility and balance gains may support positional versatility and stability [20,21,22,23,24,25]. Balance, or the ability to regain balance following a perturbation, is valued across most sports disciplines. In BJJ, it plays an important role during both standing exchanges (e.g., securing technical points through takedowns) and ground fighting (e.g., sweeps and positional control). Flexibility contributes to a greater range of technical functionality and may facilitate the execution and mastery of complex techniques, such as the berimbolo. Nevertheless, these outcomes were not directly linked to competitive performance indicators in the present study; therefore, future research should examine whether such changes translate into match performance, technical efficiency, fatigue resistance, or reduced performance decline across repeated bouts.

The observed co-occurrence of shorter training experience and greater motor changes suggested that, in most cases, participants with less training experience achieved larger training-related gains. This was particularly evident in strength and strength-endurance tests and indicators. This phenomenon may be related to the fact that these athletes had a greater potential for improvement in conditioning abilities, whereas in more experienced athletes these variables were already highly developed and therefore more difficult to further enhance [40]. This suggests that, for athletes with greater training advancement, achieving greater progression may require the use of higher-intensity exercise content, longer exposure periods, or a combination of these variables—pointing to further directions for research exploration. Interestingly, flexibility and static balance tests were exceptions within the demonstrated relationships, showing the opposite trend: athletes with longer training experience achieved greater effects of the intervention. Driven by this observation, an interview was conducted with these participants, during which they reported that they had previously neglected these training components (limited exposure to balance and flexibility exercises) and expressed strong enthusiasm for their inclusion in the mesocycle. According to the GQ protocol (see methodology), flexibility exercises were consistently implemented both in the initial and final parts of the training session, and balance-related exercises were incorporated in the circuits of the main section. This observed pattern may be explained by two co-occurring mechanisms. First, the greater improvement may have resulted from a more efficient neuromuscular system in athletes with longer training experience, which facilitated better adaptation to new training stimuli [40]. Second, athletes with greater training seniority may have previously focused mainly on developing strength, power, or endurance (as confirmed by the interview), while relatively neglecting elements such as flexibility and balance. Consequently, the introduction of a programme targeting these abilities acted as a new and potent adaptive stimulus. The bodies of advanced athletes may have been more sensitive and responsive to the intervention, generating a higher rate of progression than in less experienced individuals.

The motor adaptations observed with the use of GQ are closely linked to the optimisation of the structural profile of the studied BJJ athletes [59]. The present work forms part of the project “Grappler Quest Trilogy.” One key question remains to be addressed: How did GQ influence the multidimensional sport-specific fitness of the participants? This construct reflects a hybrid of motor abilities, exercise capacities, and technical–tactical skills [6,7]. Exploring this issue constitutes the next stage of our research.

### Limitations of the Study

This study has several limitations that should be considered when interpreting the findings. First, the randomised trial included a relatively small sample (*n* = 22 per group), which limits the precision of effect estimates and reduces sensitivity to small-to-moderate effects. Second, a broad set of motor outcomes was analysed, increasing the risk of false-positive findings due to multiple testing; therefore, multiplicity was addressed using the Benjamini–Hochberg false discovery rate procedure, and secondary outcomes should be interpreted cautiously with emphasis on effect sizes and uncertainty. Third, although two primary outcomes were prespecified, the remaining variables should be regarded as secondary or exploratory. Fourth, the analyses examining associations between training experience and intervention effects were exploratory and correlational and should be treated as hypothesis-generating rather than causal. Fifth, HR-based load monitoring was conducted during a standardised post-intervention reconstruction of the training protocol rather than during the original intervention period. Although the reconstruction followed the same procedure, these measures provide only an approximate characterisation of physiological internal load. Sixth, the study design does not allow a clear distinction between effects attributable to the specific exercise content of GQ and effects attributable to the addition of a structured conditioning block. Therefore, the findings should be interpreted as the effect of the entire GQ intervention package rather than the isolated effect of individual program components. Seventh, blinding of coaches and evaluators was not fully feasible. Eighth, a baseline imbalance was observed for simple reaction time in favour of the EXP group, increasing uncertainty around coordination-related interpretations. Finally, the sample consisted exclusively of competitive adult male Polish BJJ athletes with substantial training experience. Consequently, the findings should not be generalised without caution to female athletes, youth athletes, masters athletes, recreational practitioners, beginners, or athletes from other competitive levels and cultural training environments. Future studies should include larger and more diverse samples, direct injury and performance surveillance, and more comprehensive monitoring of external and internal training loads. In addition, no clinical endpoints were assessed, including injury incidence, injury severity, rehabilitation progress, or return-to-play outcomes. This limits the clinical interpretation of the findings and restricts any injury-related conclusions to theoretical and hypothesis-generating statements.

## 5. Conclusions

Our findings indicate that the Grappler Quest (GQ) training strategy, implemented as a structured station- and circuit-based intervention, was associated with greater improvements in the motor fitness profile of competitive Brazilian Jiu-Jitsu athletes than standard training alone. The most robust findings concerned the prespecified primary strength-endurance outcomes, namely the bench press and squat performed with 50% body mass load. Improvements were also observed across several secondary motor-performance outcomes, including selected strength, grip-endurance, flexibility, and balance indicators; however, these results should be interpreted with caution because of the number of outcomes assessed.

The findings support the potential usefulness of incorporating structured circuit-based conditioning into BJJ preparation, particularly during phases aimed at developing strength endurance and general motor readiness. However, the present design does not allow the specific exercise content of GQ to be separated from the broader effect of adding a structured conditioning block to standard practice. Therefore, the results should be interpreted as evidence for the effectiveness of the overall GQ intervention package rather than proof of superiority of the specific GQ methodology over other conditioning approaches.

Exploratory analyses suggested that shorter training experience was generally associated with larger conditioning-related improvements, whereas flexibility and balance showed the opposite pattern. These associations are correlational and should be confirmed in future studies designed to examine moderators of training response. No injury incidence, injury severity, rehabilitation, or return-to-play endpoints were assessed; therefore, any implications for athlete health, injury prevention, or return to training remain indirect and hypothesis-generating. Larger studies involving more diverse populations, direct performance indicators, prospective injury surveillance, and comprehensive internal and external load monitoring are required to confirm and extend these findings.

### Practical Implications

The present findings have practical relevance for BJJ coaches, athletes, and practitioners involved in athlete preparation and health monitoring. The GQ framework may be considered as a complementary conditioning method within the preparatory phase or selected blocks of a periodized training cycle, particularly when the training goal is to improve strength endurance, grip-related endurance, flexibility, balance, and general motor readiness. The protocol should not be interpreted as a medical, rehabilitation, or injury-prevention intervention. In practical terms, it may be implemented alongside regular technical–tactical training and sparring, provided that total training exposure, fatigue, and recovery are monitored.

Coaches should treat GQ as an adjunct rather than a replacement for technical BJJ practice. The exercise content should be individualised according to training age, belt level, body mass, injury history, and current phase of preparation. For less experienced competitive athletes, the emphasis may be placed on movement quality, basic strength endurance, and progressive exposure to circuit density. For more experienced athletes, progression may require more careful manipulation of task complexity, work duration, external load, and recovery intervals. The expected practical outcomes should be framed primarily as improvements in motor fitness components rather than direct evidence of improved match performance or reduced injury risk.

The GQ framework may also be adapted to other combat sports, particularly grappling-based disciplines, such as judo, wrestling, sambo, and MMA. However, transfer to other sports should not be automatic. Exercise selection, movement patterns, work–rest structure, and loading parameters should be modified according to the technical and physiological demands of each discipline. Further studies are needed to confirm the feasibility and effectiveness of such adaptations.

## Figures and Tables

**Table 1:** Statistical characteristics of basic somatic traits, age, and training experience in the examined groups of BJJ athletes (EXP vs. CON).

Variables	Group EXP (*n* = 22); $\tilde{x}$ ± sd	Group CON (*n* = 22); $\tilde{x}$ ± sd	*p*-Value
Age [years]	26.02 ± 3.24	25.59 ± 3.25	0.597
Body height [cm]	177.34 ± 6.33	176.90 ± 6.22	0.819
Body mass [kg]	78.79 ± 12.05	77.88 ± 9.86	0.879
BMI [kg/m^2^]	24.98 ± 3.04	24.86 ± 2.71	0.894
Training experience [years]	7.50 ± 2.67	7.64 ± 2.79	0.879

$\tilde{x}$—arithmetic mean; sd—standard deviation; *p*—level of significance.

**Table 2:** Experimental training program—methodological framework.

Experimental Training Program Based on the Circuit Method	
Parameter	Value/Description
Number of exercise circuits	3
Number of exercises per circuit	5
Repetitions/Duration per exercise	Circuit I: 8 to 15 reps/Circuit II and III: 30 s
% of maximum load	Up to 50% of body mass
Exercise tempo	Circuit I: 2-1-2-1/Circuit II and III: fast-paced
Rest time between circuits	180 s

**Table 3:** Types of exercises used in the experimental program.

I Strength–Endurance Circuit	II Functional Circuit	III Specialized Circuit
Pull-ups on a suspended gi to full elbow flexion and extension	Wall roll ^1^	BJJ Burpees ^5^ across the length of the training hall
Classic squat combined with a medicine ball throw over the head and then catching it	Knee-on-belly ^2^ with side-to-side switching (R-L) on a training bag	Sit-up from the back position into a kimura setup ^6^
Trunk twists with a plate held in hands, elbows bent at chest height	Minimum forward and backward rolls on a gymnastic ball (or other exercises that are not a technical barrier, e.g., handstands, clean and press, rolls, etc.)	Isometric hold of steel maces at a 90-degree elbow angle
Deadlift with kettlebells, pulling the weight up to the clavicle line (hands close to the torso)	BJJ floor climb ^3^ forward and backward across the training hall	Stable standing on a gymnastic ball with feet or knees, no hands used for support
Deep push-ups on parallel bars	Gymnastic exercise set ^4^ across the training hall with fluid motion	Jump over a rope (1-m height) followed by crawling under it

^1^ Wall roll—A specialised BJJ exercise with a specific movement pattern (natural gymnastics) [37]. ^2^ Knee-on-belly—A ground position scored in BJJ [37]. ^3^ BJJ floor climb—A specialised BJJ exercise with a specific movement pattern (natural gymnastics) [37]. ^4^ Gymnastic exercise set—Composed of the following elements: T1. Forward roll; T2. Headstand with muscle power and self-support by forward roll; T3. Handstand with self-support by forward roll; T4. Direction change with a jump and a half-turn (180 degrees); T5. Backward roll. ^5^ BJJ Burpees—An intense exercise engaging all muscle groups. One sequence consists of 4 movements performed one after the other: T1. Forward torso bend with hands on the floor; T2. Hand transition to a front support position; T3. Arm bend in front support; T4. Jumping transition to a supported squat; T5. Jumping upwards [37]. ^6^ Kimura—from a supine position, transition to a seated position with torso rotation to simulate the kimura lock (solo drill) or execute the shoulder lock technique (with a partner) [37].

**Table 4:** ANCOVA analysis of post-intervention outcomes: Comparison of EXP group (*n* = 22) vs. CON group (*n* = 22) in BJJ athletes (*n* = 44).

Variables	Group CON Adjusted Post (95% CI)	Group EXP Adjusted Post (95% CI)	F (df1, df2)	*p*	η^2^
Static handgrip strength [kG]	49.68 (49.16–50.20)	51.74 (51.22–52.27)	31.66 (1, 41)	<0.001 **	0.44 ^S^
RS_SH_ [dq]	0.65 (0.64–0.66)	0.69 (0.68–0.69)	28.89 (1, 41)	<0.001 **	0.33 ^S^
Bench press 1RM [kg]	89.74 (88.33–91.15)	93.67 (92.26–95.08)	15.87 (1, 41)	<0.001 **	0.28 ^S^
RS_BP_ [dq]	1.16 (1.14–1.17)	1.21 (1.19–1.23)	17.24 (1, 41)	<0.001 **	0.30 ^S^
Squat 1RM [kg]	109.22 (107.89–110.55)	112.60 (111.27–113.93)	13.13 (1, 41)	<0.001 **	0.24 ^S^
RS_S_ [dq]	1.40 (1.38–1.42)	1.45 (1.43–1.47)	13.18 (1, 41)	<0.01 *	0.24 ^S^
Pull ups in judogi [reps]	13.84 (13.35–14.32)	15.57 (15.08–16.06)	25.8 (1, 41)	<0.001 **	0.39 ^S^
RS_PU_ [dq]	0.19 (0.16–0.22)	0.20 (0.17–0.23)	9.58 (1, 41)	<0.01 *	0.19 ^S^
Flexed-arm hang in judogi [s]	41.53 (40.54–42.51)	44.53 (43.41–45.51)	18.92 (1, 41)	<0.001 **	0.32 ^S^
RS_H1_ [dq]	0.54 (0.52–0.55)	0.58 (0.57–0.59)	19.89 (1, 41)	<0.001 **	0.33 ^S^
Straight-arm hang in judogi [s]	53.1 (52.01–54.18)	56.8 (55.72–57.89)	30.87 (1, 41)	<0.001 **	0.43 ^S^
RS_H2_ [dq]	0.70 (0.68–0.71)	0.75 (0.73–0.76)	22.19 (1, 41)	<0.001 **	0.35 ^S^
Sit-ups [reps in 30 s]	33.1 (32.57–33.64)	33.49 (32.95–34.02)	1.02 (1, 41)	0.32	0.12 ^M^
Back extensions [reps in 30 s]	49.7 (49.2–50.2)	50.3 (49.8–50.8)	3.01 (1, 41)	0.09	0.07 ^M^
Bench press 50% BM [reps]	38.84 (38.48–39.21)	40.57 (40.29–40.93)	27.34 (1, 41)	<0.001 **	0.52 ^S^
Squat 50% BM [reps]	41.35 (40.87–42.01)	43.78 (43.12–44.44)	27.7 (1, 41)	<0.001 **	0.40 ^S^
Shuttle run 10 × 5 m [s]	20.28 (20.11–20.45)	20.02 (19.85–20.19)	4.97 (1, 41)	0.03 *	0.11 ^M^
Standing long jump [cm]	229.76 (228.73–230.78)	230.84 (229.81–231.87)	2.24 (1, 41)	0.14	0.05
Static balance [s]	13.82 (12.3–15.34)	17.76 (16.24–19.28)	13.64 (1, 41)	<0.001 *	0.25 ^S^
Simple reaction time [s]	0.24 (0.23–0.24)	0.24 (0.23–0.24)	0.002 (1, 41)	0.97	0.00
Trunk flexibility [cm]	28.25 (27.67–28.82)	30.75 (30.18–31.33)	38.88 (1, 41)	<0.001 *	0.49 ^S^
Maximal straddle sitting position [cm]	48.39 (47.68–49.1)	45.93 (45.22–46.64)	24.58 (1, 41)	<0.001 *	0.37 ^S^

Adjusted post—adjusted mean, results adjusted for the influence of confounding variables (covariates); 95% CI—95% confidence intervals; F (df1, df2)—F statistic and degrees of freedom; dq—dimensionless quantity; RS [Relative Strength]: _SH_—static handgrip; _BP_—bench press; _S_—squat; _PU_—pull ups; _H1_—flexed-arm hang; _H2_—straight-arm hang; *p*—level of significance for differentiation; η^2^—eta squared, indicating effect size; * statistically significant values (*p* < 0.05); ** statistically significant values (*p* < 0.001); ^M^ moderate effect size; ^S^ strong effect size.

**Table 5:** Statistical characteristics of the motor profile and their intragroup variation in the studied groups (EXP *n* = 22; CON *n* = 22) of BJJ athletes (*n* = 44).

Variables	Measurement	Group EXP (*n* = 22)					Group CON (*n* = 22)				
		Mean	SD	CV%	*p*	*d_C_*	Mean	SD	CV%	*p*	*d_C_*
Static handgrip strength [kG]	I pre-test	48.66	9.95	20.45	<0.001 **	0.29	49.65	9.93	20.01	0.125	0.05
	II post-test	51.30	8.48	16.53			50.12	9.32	18.59		
RS_SH_ [dq]	I pre-test	0.64	0.19	30.30	<0.001 **	0.22	0.65	0.19	28.48	0.149	0.05
	II post-test	0.68	0.18	26.68			0.66	0.18	26.95		
Bench press 1RM [kg]	I pre-test	88.09	14.34	16.28	<0.001 **	0.37 ^M^	88.82	14.39	16.21	0.006 *	0.09
	II post-test	93.32	13.72	14.71			90.09	14.77	16.40		
RS_BP_ [dq]	I pre-test	1.13	0.14	12.71	<0.001 **	0.54 ^M^	1.15	0.16	13.93	0.001 *	0.13
	II post-test	1.20	0.12	9.93			1.17	0.16	14.14		
Squat 1RM [kg]	I pre-test	107.41	22.53	20.98	0.001 *	0.20	109.32	22.66	20.73	0.287	0.04
	II post-test	111.68	20.86	18.68			110.14	22.84	20.74		
RS_S_ [dq]	I pre-test	1.36	0.18	13.06	<0.001 **	0.42 ^M^	1.40	0.20	13.92	0.123	0.10
	II post-test	1.43	0.15	10.35			1.42	0.21	14.55		
Pull ups in judogi [reps]	I pre-test	13.36	5.13	38.40	<0.001 **	0.39 ^M^	14.05	4.77	33.93	0.628	0.02
	II post-test	15.27	4.58	29.98			14.14	4.40	31.13		
RS_PU_ [dq]	I pre-test	0.18	0.08	44.72	<0.001 **	0.27	0.19	0.07	39.37	0.454	0.00
	II post-test	0.20	0.07	35.76			0.19	0.07	37.80		
Flexed-arm hang in judogi [s]	I pre-test	40.21	9.83	24.45	<0.001 **	0.40 ^M^	41.07	12.91	31.43	0.022 *	0.07
	II post-test	44.12	9.53	21.59			41.94	12.49	29.79		
RS_H1_ [dq]	I pre-test	0.52	0.13	25.23	<0.001 **	0.40 ^M^	0.54	0.19	34.69	0.024 *	0.05
	II post-test	0.57	0.12	20.95			0.55	0.18	33.14		
Straight-arm hang in judogi [s]	I pre-test	51.72	13.14	25.40	<0.001 **	0.40 ^M^	52.12	13.38	25.67	0.008 *	0.09
	II post-test	56.63	11.61	20.50			53.27	12.51	23.48		
RS_H2_ [dq]	I pre-test	0.67	0.23	33.68	<0.001 **	0.32	0.68	0.23	33.16	0.002 *	0.09
	II post-test	0.74	0.21	27.80			0.70	0.22	31.76		
Sit-ups [reps in 30 s]	I pre-test	32.05	3.67	11.45	0.001 *	0.29	32.91	4.44	13.49	0.049 *	0.14
	II post-test	33.09	3.58	10.83			33.50	4.24	12.66		
Back extensions [reps in 30 s]	I pre-test	49.27	5.95	12.07	0.005 *	0.14	49.73	5.36	10.77	0.433	0.04
	II post-test	50.09	5.90	11.79			49.91	4.90	9.81		
Bench press 50% BM [reps]	I pre-test	38.36	4.09	10.66	<0.001 **	0.51 ^M^	39.05	4.31	11.05	0.687	0.02
	II post-test	40.27	3.34	8.30			39.14	4.06	10.38		
Squat 50% BM [reps]	I pre-test	40.95	6.12	14.95	<0.001 **	0.44 ^M^	41.59	4.98	11.98	0.892	0.01
	II post-test	43.50	5.55	12.77			41.64	4.77	11.45		
Shuttle run 10 × 5 m [s]	I pre-test	19.99	1.16	5.81	0.559	0.10	20.31	1.22	5.99	0.445	0.09
	II post-test	19.88	1.00	5.81			20.42	1.19	5.84		
Standing long jump [cm]	I pre-test	228.77	13.02	5.69	0.001 *	0.12	229.95	11.05	4.80	0.671	0.03
	II post-test	230.32	12.24	5.31			230.27	9.22	4.00		
Static balance [s]	I pre-test	14.22	10.57	74.30	<0.001 **	0.39 ^M^	12.24	9.26	75.69	0.168	0.06
	II post-test	18.81	12.97	68.96			12.77	8.88	69.55		
Simple reaction time [s]	I pre-test	0.23	0.02	8.43	0.514	0.00	0.25	0.01	5.45	0.208	1.00 ^S^
	II post-test	0.23	0.02	8.72			0.24	0.01	5.21		
Trunk flexibility [cm]	I pre-test	28.68	6.61	23.04	<0.001 **	0.43 ^M^	27.18	5.93	21.80	0.246	0.05
	II post-test	31.50	6.64	21.08			27.50	6.09	22.15		
Maximal straddle sitting position [cm]	I pre-test	48.64	3.58	7.36	<0.001 **	0.76 ^S^	49.05	3.97	8.09	0.078	0.12
	II post-test	45.73	4.06	8.88			48.59	4.02	8.27		

SD—standard deviation; CV—coefficient of variation; dq—dimensionless quantity; RS [Relative Strength]: _SH_—static handgrip; _BP_—bench press; _S_—squat; _PU_—pull ups; _H1_—flexed-arm hang; _H2_—straight-arm hang; *p*—level of significance for differentiation; *d_c_*—Cohen effect size for differentiation; * statistically significant values (*p* < 0.05); ** statistically significant values (*p* < 0.001); ^M^ moderate effect size; ^S^ strong effect size.

**Table 6:** Correlation coefficients between training experience and training effects (delta) in the EXP group.

Variables	Group EXP (*n* = 22)	
	r_s_	*p*
Static handgrip strength [kG]	−0.79	<0.001 **
RS_SH_ [dq]	−0.75	<0.001 **
Bench press 1RM [kg]	−0.68	0.001 *
RS_BP_ [dq]	−0.70	<0.001 **
Squat 1RM [kg]	−0.45	0.037 *
RS_S_ [dq]	−0.46	0.030 *
Pull ups in judogi [reps]	−0.76	<0.001 **
RS_PU_ [dq]	−0.74	<0.001 **
Flexed-arm hang in judogi [s]	−0.74	<0.001 **
RS_H1_ [dq]	−0.73	<0.001 **
Straight-arm hang in judogi [s]	−0.83	<0.001 **
RS_H2_ [dq]	−0.82	<0.001 **
Sit-ups [reps in 30 s]	−0.76	<0.001 **
Back extensions [reps in 30 s]	−0.47	0.027 *
Bench press 50% BM [reps]	−0.77	<0.001 **
Squat 50% BM [reps]	−0.72	<0.001 **
Shuttle run 10 × 5 m [s]	0.25	0.311
Standing long jump [cm]	−0.77	<0.001 **
Static balance [s]	0.53	0.012 *
Simple reaction time [s]	0.23	0.321
Trunk flexibility [cm]	0.50	0.018 *
Maximal straddle sitting position [cm]	−0.48	0.021 *

r_s_—value of the correlation coefficient Spearman; dq—dimensionless quantity; RS [Relative Strength]: _SH_—static handgrip; _BP_—bench press; _S_—squat; _PU_—pull ups; _H1_—flexed-arm hang; _H2_—straight-arm hang; *p*—level of significance; * a statistically significant level of differentiation (*p* < 0.05); ** statistically significant values (*p* < 0.001).

## Data Availability

The data presented in this study are available upon request from the corresponding author.

## Supplementary Materials

The following supporting information can be downloaded at: https://drive.google.com/file/d/1r6upRR6qWIZMQUaGzbvrpblGApuv17Xy/view?usp=sharing (accessed on 28 June 2026), Video S1: Instructional and training video presenting the Grappler Quest station-based circuit structure, exercise setup, execution cues, and work–rest organisation. The video will be archived as supplementary material by the journal to support long-term accessibility and reproducibility of the intervention.

## Appendix A

**Table A1:** Statistical characteristics of the motor profile and their intergroup variation in the studied groups (EXP *n* = 22 vs. CON *n* = 22) of BJJ athletes (*n* = 44).

Measurement	Group EXP (*n* = 22)					Group CON (*n* = 22)					d	*p*	*d_C_*
	$\tilde{x}$ ± sd	Me	Min	Max	CV%	$\tilde{x}$ ± sd	Me	Min	Max	CV%			
Static handgrip strength [kG]													
I pre-test	48.7 ± 10.0	46.1	33.9	68.2	20.5	49.7 ± 9.9	47.5	35.9	71.3	20.0	−1.0	0.744	0.10
II post-test	51.3 ± 8.5	49.3	38.2	69.0	16.5	50.1 ± 9.3	49.4	35.6	70.2	18.6	1.2	0.661	0.13
RS_SH_ [dq]													
I pre-test	0.6 ± 0.2	0.6	0.4	1.0	30.3	0.7 ± 0.2	0.6	0.4	1.0	28.5	−0.1	0.565	0.50 ^M^
II post-test	0.7 ± 0.2	0.7	0.4	1.0	26.7	0.7 ± 0.2	0.6	0.4	1.0	27.0	0.0	0.715	0.00
Bench press 1RM [kg]													
I pre-test	88.1 ± 14.3	85.0	71.0	130.0	16.3	88.8 ± 14.4	86.5	72.0	135.0	16.2	−0.7	0.796	0.05
II post-test	93.3 ± 13.7	90.0	72.0	136.0	14.7	90.1 ± 14.8	88.0	72.0	138.0	16.4	3.2	0.405	0.22
RS_BP_ [dq]													
I pre-test	1.1 ± 0.1	1.1	0.9	1.5	12.7	1.2 ± 0.2	1.1	0.9	1.4	13.9	−0.1	0.644	0.67 ^S^
II post-test	1.2 ± 0.1	1.2	1.0	1.5	9.9	1.2 ± 0.2	1.1	0.9	1.5	14.1	0.0	0.390	0.00
Squat 1RM [kg]													
I pre-test	107.4 ± 22.5	100.0	80.0	160.0	21.0	109.3 ± 22.7	102.5	86.0	165.0	20.7	−1.9	0.664	0.08
II post-test	111.7 ± 20.9	103.0	88.0	160.0	18.7	110.1 ± 22.8	102.5	85.0	165.0	20.7	1.6	0549	0.07
RS_S_ [dq]													
I pre-test	1.4 ± 0.2	1.4	1.0	1.7	13.1	1.4 ± 0.2	1.5	1.1	1.7	13.9	0.0	0.501	0.00
II post-test	1.4 ± 0.2	1.5	1.2	1.7	10.4	1.4 ± 0.2	1.5	1.1	1.7	14.6	0.0	0.760	0.00
Pull ups in judogi [lp]													
I pre-test	13.4 ± 5.1	12.5	6.0	26.0	38.4	14.1 ± 4.8	13.5	8.0	25.0	33.9	−0.7	0.650	0.14
II post-test	15.3 ± 4.6	15.5	8.0	27.0	30.0	14.1 ± 4.4	13.5	8.0	24.0	31.1	1.2	0.406	0.27
RS_PU_ [dq]													
I pre-test	0.2 ± 0.1	0.2	0.1	0.4	44.7	0.2 ± 0.1	0.2	0.1	0.4	39.4	0.0	0.665	0.00
II post-test	0.2 ± 0.1	0.2	0.1	0.4	35.8	0.2 ± 0.1	0.2	0.1	0.3	37.8	0.0	0.526	0.00
Flexed-arm hang in judogi [s]													
I pre-test	40.2 ± 9.8	40.0	25.1	60.6	24.5	41.1 ± 12.9	43.3	26.1	71.1	31.4	−0.9	0.888	0.08
II post-test	44.1 ± 9.5	45.6	28.0	64.1	21.6	41.9 ± 12.5	43.2	27.1	69.1	29.8	2.2	0.518	0.20
RS_H1_ [dq]													
I pre-test	0.5 ± 0.1	0.5	0.3	0.8	25.2	0.5 ± 0.2	0.6	0.3	0.9	34.7	0.0	0.661	0.00
II post-test	0.6 ± 0.1	0.5	0.4	0.8	21.0	0.6 ± 0.2	0.5	0.3	0.9	33.1	0.0	0.654	0.00
Straight-arm hang in judogi [s]													
I pre-test	51.7 ± 13.1	50.3	32.4	78.0	25.4	52.1 ± 13.4	50.9	33.0	81.2	25.7	−0.4	0.921	0.03
II post-test	56.6 ± 11.6	56.6	37.4	82.1	20.5	53.3 ± 12.5	51.7	35.0	82.4	23.5	3.3	0.362	0.27
RS_H2_ [dq]													
I pre-test	0.7 ± 0.2	0.7	0.4	1.3	33.7	0.7 ± 0.2	0.6	0.4	1.3	33.2	0.0	0.842	0.00
II post-test	0.7 ± 0.2	0.7	0.5	1.4	27.8	0.7 ± 0.2	0.7	0.5	1.3	31.8	0.0	0.296	0.00
Sit-ups [reps in 30 s]													
I pre-test	32.1 ± 3.7	32.5	26.0	38.0	11.5	32.9 ± 4.4	33.5	22.0	39.0	13.5	−0.8	0.318	0.20
II post-test	33.1 ± 3.6	33.5	27.0	38.0	10.8	33.5 ± 4.2	34.0	23.0	40.0	12.7	−0.4	0.731	0.10
Back extensions [reps in 30 s]													
I pre-test	49.3 ± 6.0	50.0	36.0	57.0	12.1	49.7 ± 5.4	50.0	38.0	56.0	10.8	−0.4	0.769	0.07
II post-test	50.1 ± 5.9	51.0	37.0	57.0	11.8	49.9 ± 4.9	51.0	39.0	55.0	9.8	0.2	0.734	0.04
Bench press 50% BM [reps]													
I pre-test	38.4 ± 4.1	38.0	30.0	45.0	10.7	39.1 ± 4.3	39.5	29.0	45.0	11.1	−0.7	0.593	0.17
II post-test	40.3 ± 3.3	40.0	32.0	45.0	8.3	39.1 ± 4.1	39.5	30.0	44.0	10.4	1.2	0.318	0.32
Squat 50% BM [reps]													
I pre-test	41.0 ± 6.1	40.0	31.0	52.0	15.0	41.6 ± 5.0	41.0	32.0	49.0	12.0	−0.6	0.707	0.11
II post-test	43.5±5.6	43.5	33.0	53.0	12.8	41.6 ± 4.8	40.5	33.0	49.0	11.5	1.9	0.239	0.37 ^M^
Shuttle run 10 × 5 m [s]													
I pre-test	20.0 ± 1.2	19.5	18.5	22.3	5.8	20.3 ± 1.2	19.9	19.0	22.5	6.0	−0.3	0.392	0.25
II post-test	19.9 ± 1.0	19.8	18.6	22.3	5.0	20.4 ± 1.2	20.1	19.0	22.5	5.8	−0.5	0.127	0.45 ^M^
Standing long jump [cm]													
I pre-test	228.8 ± 13.0	232.0	209.0	258.0	5.7	230.0 ± 11.1	229.5	208.0	256.0	4.8	−1.2	0.747	0.10
II post-test	230.3 ± 12.2	233.0	212.0	256.0	5.3	230.3 ± 9.2	229.0	210.0	254.0	4.0	0.0	0.989	0.00
Static balance [s]													
I pre-test	14.2 ± 10.6	10.2	3.6	35.4	74.3	12.2 ± 9.3	8.3	2.8	32.1	75.7	2.0	0.467	0.20
II post-test	18.8 ± 13.0	11.0	5.1	42.3	69.0	12.8 ± 8.9	9.1	4.1	35.2	69.6	6.0	0.064	0.55 ^M^
Simple reaction time [s]													
I pre-test	0.23 ± 0.02	0.2	0.2	0.3	8.4	0.25 ± 0.01	0.3	0.2	0.3	5.5	0.02	0.003 *	1.33 ^S^
II post-test	0.23 ± 0.02	0.2	0.2	0.3	8.7	0.24 ± 0.01	0.2	0.2	0.3	5.2	0.01	0.002 *	0.67 ^S^
Trunk flexibility [cm]													
I pre-test	28.7 ± 6.6	26.5	18.0	40.0	23.0	27.2 ± 5.9	28.0	15.0	38.0	21.8	1.5	0.432	0.24
II post-test	31.5 ± 6.6	30.0	21.0	42.0	21.1	27.5 ± 6.1	27.5	15.0	39.0	22.2	4.0	0.043 *	0.63 ^M^
Maximal straddle sitting position [cm]													
I pre-test	48.6 ± 3.6	49.0	43.0	55.0	7.4	49.1 ± 4.0	47.0	44.0	58.0	8.1	−0.5	0.879	0.13
II post-test	45.7 ± 4.1	46.0	39.0	57.0	8.9	48.6 ± 4.0	47.0	44.0	56.0	8.3	−2.9	0.049 *	0.72 ^S^

$\tilde{x}$—arithmetic mean; Me—median; sd—standard deviation; min—minimum value; max—maximum value; CV%—coefficient of variation; dq—dimensionless quantity; I—first measurement period (pre-test); II—second measurement period (post-test); RS [Relative Strength] _SH_—static handgrip; _BP_—bench press; _S_—squat; _PU_—pull ups; _H1_—flexed-arm hang; _H2_—straight-arm hang; d—difference between means (delta); *p*—level of significance; * a statistically significant level of differentiation (*p* < 0.05); *d_C_*—effect size expressed using Cohen’s *d* coefficient; ^M^—moderate effect; ^S^—strong effect.

The coefficients of variation demonstrated that the internal variability of most analysed test variables was very low in both groups (CV < 25%) for both the pre-test and post-test assessments. The highest level of heterogeneity was observed for the static balance test, where CV > 45%, indicating substantial variability. The averaged CV% values from both measurement sessions show that the within-group variability of performance in the pre-test was lower in the CON group (mean test index: EXP = 20.00 vs. CON = 19.83). In the post-test, an opposite trend was noted, with greater improvement in homogeneity in the EXP group (EXP = 17.73 vs. CON = 18.73) (Appendix A—Table A1).

**Table A2:** Benjamini–Hochberg FDR adjustment for ANCOVA *p*-values (based on Table 4). Note: For entries reported as *p* < 0.001 or *p* < 0.01 in Table 4, the respective upper-bound values (0.001 or 0.01) were used to compute conservative q-values.

Variables	*p* (ANCOVA; Unadjusted)	q (BH-FDR)	FDR-Significant (q < 0.05)
Static handgrip strength [kG]	<0.001 **	≤0.0015	Yes
RS_SH_ [dq]	<0.001 **	≤0.0015	Yes
Bench press 1RM [kg]	<0.001 **	≤0.0015	Yes
RS_BP_ [dq]	<0.001 **	≤0.0015	Yes
Squat 1RM [kg]	<0.001 **	≤0.0015	Yes
RS_S_ [dq]	<0.01 *	≤0.0129	Yes
Pull ups in judogi [reps]	<0.001 **	≤0.0015	Yes
RS_PU_ [dq]	<0.01 *	≤0.0129	Yes
Flexed-arm hang in judogi [s]	<0.001 **	≤0.0015	Yes
RS_H1_ [dq]	<0.001 **	≤0.0015	Yes
Straight-arm hang in judogi [s]	<0.001 **	≤0.0015	Yes
RS_H2_ [dq]	<0.001 **	≤0.0015	Yes
Sit-ups [reps in 30 s]	0.32	0.3352	No
Back extensions [reps in 30 s]	0.09	0.1042	No
Bench press 50% BM [reps]	<0.001 **	≤0.0015	Yes
Squat 50% BM [reps]	<0.001 **	≤0.0015	Yes
Shuttle run 10 × 5 m [s]	0.03 *	0.0367	Yes
Standing long jump [cm]	0.14	0.1540	No
Static balance [s]	<0.001 *	≤0.0015	Yes
Simple reaction time [s]	0.97	0.9700	No
Trunk flexibility [cm]	<0.001 *	≤0.0015	Yes
Maximal straddle sitting position [cm]	<0.001 *	≤0.0015	Yes

*p* (ANCOVA; unadjusted)—nominal (unadjusted) two-sided *p*-value from the ANCOVA group effect; q (BH-FDR)—*p*-value adjusted for multiple comparisons using the Benjamini–Hochberg false discovery rate (FDR) procedure; FDR-significant (q < 0.05)—indicates whether the result remains statistically significant after FDR adjustment (q < 0.05); dq—dimensionless quantity; RS [Relative Strength]: _SH_—static handgrip; _BP_—bench press; _S_—squat; _PU_—pull ups; _H1_— flexed-arm hang; _H2_—straight-arm hang; *p*—level of significance for differentiation; * statistically significant values (*p* < 0.05); ** statistically significant values (*p* < 0.001).
